# First‐Line Treatment of Advanced Thoracic SMARCA4‐Deficient Undifferentiated Tumor: A Case Report and Review of the Literature

**DOI:** 10.1155/crom/7148051

**Published:** 2026-03-09

**Authors:** Qingyang Wen, Mengle Long, Feng Li, Yaya Peng, Jiarong Yi, Yongjun Wu, Di Wang

**Affiliations:** ^1^ Department of Oncology, Graduate Collaborative Training Base of The First People′s Hospital of Xiangtan City, Hengyang Medical School, University of South China, Hengyang, Hunan, China, usc.edu.cn; ^2^ Department of Medical Oncology, The First People′s Hospital of Xiangtan City, Xiangtan, Hunan, China; ^3^ Department of Pathology, The First People′s Hospital of Xiangtan City, Xiangtan, Hunan Province, China; ^4^ Department of Pathology, Xiangtan Center Hospital, Xiangtan City, Hunan province, China; ^5^ Department of Pathology, The Affiliated Hospital of Hunan University, Xiangtan City, Hunan Province, China

**Keywords:** antivascular therapy, case report, immunohistochemistry, immune-mediated myocarditis, thoracic SMARCA4-deficient undifferentiated tumor

## Abstract

**Background:**

Thoracic SMARCA4‐deficient undifferentiated tumor (SMARCA4‐UT) of the chest is a highly aggressive smoking‐related thoracic malignancy with a median overall survival (OS) of only 4–7 months.

**Case Presentation:**

In this article, we report a case of a 74‐year‐old male patient who was admitted to the hospital with recurrent cough with sputum and CT suggestive of a right pleural occupying lesion. Admission to the hospital and perfect CT showed right pleural thickening with multiple metastases in the mediastinum and liver, and the diagnosis of SMARCA4‐UT (SMARCA4 expression deletion) was confirmed by pathologic biopsy and immunohistochemistry. The genetic test map suggested high PD‐L1 expression (TPS 80%) and the patient was treated with sindilizumab (200 mg q3w) combined with bevacizumab (500 mg q3w). Grade 3 immune myocarditis occurred during treatment, and bevacizumab maintenance therapy was continued after discontinuing immunosuppression. During follow‐up, the patient achieved a final OS of 18 months.

**Conclusions:**

This case suggests that PD‐1 inhibitors combined with antiangiogenic therapy may improve the prognosis of SMARCA4‐UT, but the adverse effects observed during the treatment course demanded equally critical attention.

## 1. Introduction

SMARCA4, mapped to the 19p13 chromosomal region, encodes BRG1—an essential ATP‐dependent catalytic subunit within the SWI/SNF chromatin remodeling complex responsible for nucleosome repositioning [1–3]. This complex is involved in cell cycle progression and DNA damage repair by regulating gene transcriptional activity [4, 5]. BRG1 has been identified as a type of oncogene, with inactivating mutations occurring in approximately 16%–20% of cases [6]. Furthermore, these mutations have been reported in a variety of solid malignant tumors, including ovarian hypercalcemic small‐cell carcinoma, non–small‐cell lung cancer, medulloblastoma, and breast cancer [7–9].

The earliest discovery of the disease was made in 2015 by Loarer et al. [10]. The World Health Organization Classification of Tumors 2021 officially classifies it as a new tumor type. This tumor is most commonly found in males, with an age range of approximately 27–90 years; over 90% of patients have a history of smoking [1, 4]. It primarily involves the mediastinal region, followed by the pleura and lungs. Histologically, the tumor is usually undifferentiated or resembles rhabdomyoblasts, and it is characterized by the absence of SMARCA4 gene expression as well as local or diffuse expression of markers such as SOX2, CD34, and SALL4 [4]. Due to the aggressive nature of this tumor and the lack of effective treatments, the median survival of patients is typically 4–7 months. In this paper, we report a case of a patient with advanced SMARCA4‐deficient undifferentiated tumors (SMARCA4‐UT) who was treated with an immune checkpoint inhibitor (sindilizumab) in combination with an anti‐angiogenic drug (bevacizumab). Remarkably, this patient survived for 18 months after treatment.

## 2. Case Reports

**Figure 2:**
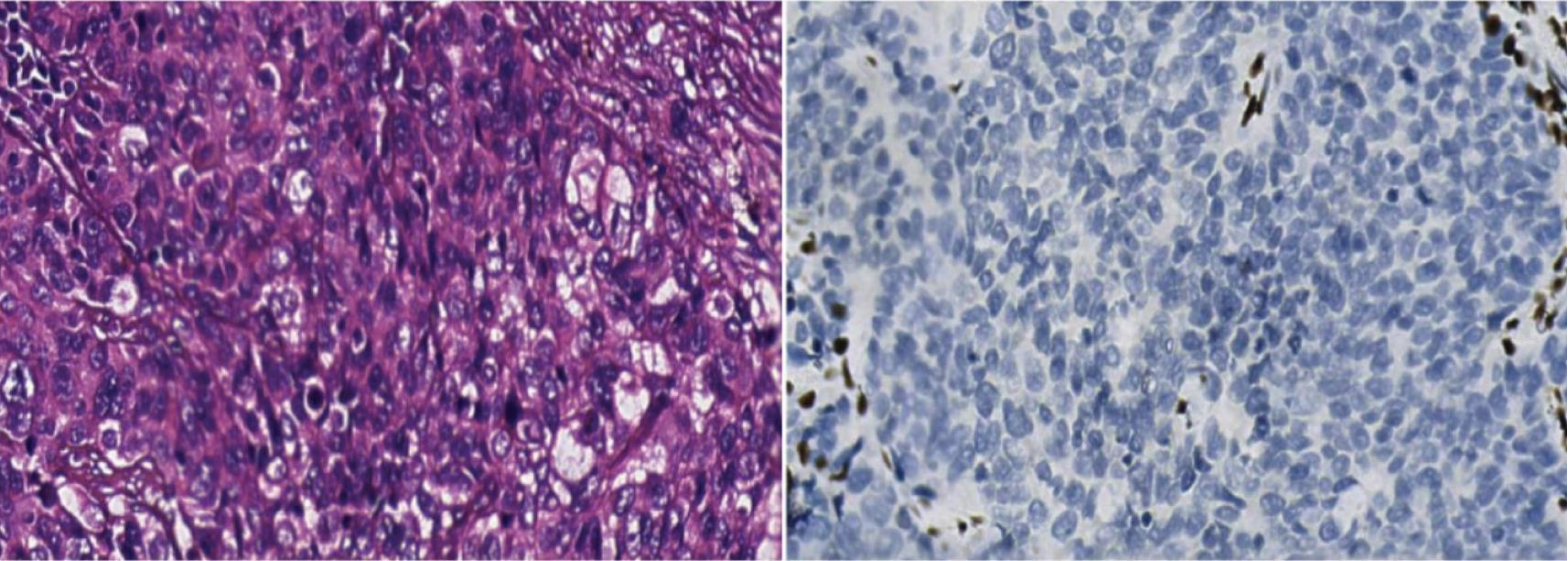
The pathological result of this patient.

The patient was a 74‐year‐old male presenting with a right pleural mass detected incidentally 1 week prior. He had a 40‐pack‐year smoking history (20–30 cigarettes/day), essential hypertension, and no family history of malignancy. Initial chest computed tomographic (CT) revealed right pleural mass lesions. Subsequent CT image (September 2023) (Figure 1) demonstrated irregular right pleural thickening (maximum 36 mm), multiple hypodense metastatic nodules in the right upper lobe (largest 26 mm), and enlarged lymph nodes in the right hilum and mediastinum (maximum 10 mm), with moderate right pleural effusion. Histopathological analysis of pleural biopsy specimens (Figure 2) showed moderately to poorly differentiated malignancy featuring medium‐sized tumor cells with active karyorrhexis and focal necrosis (30% of tumor area). Immunohistochemical profiling confirmed SMARCA4‐deficient thoracic undifferentiated tumor, demonstrating CKpan (focal weak+), CD34(+), Synaptophysin(+), STAT6(+), CAM5.2 (focal+), CK18 (focal+), INI1 retained(+), p53 overexpression(+), and SMARCA4 loss(−). Negative markers included CK7, NapsinA, CD56, ERG, TTF‐1, S‐100, calretinin, chromogranin A, INSM1, and SSTR2A. Next‐generation sequencing revealed no actionable driver mutations, whereas PD‐L1 immunohistochemistry demonstrated high expression (tumor proportion score 80%). The patient was ultimately diagnosed with Stage IV SMARCA4‐UT.

Figure 1(a) The results of a chest CT in our hospital on September 2023 and (b) the results of a chest CT in our hospital on November 2023.

**Figure:**
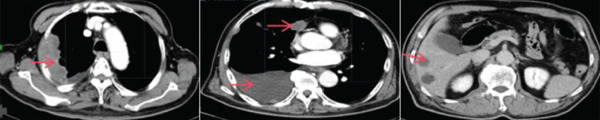
(a)

**Figure:**
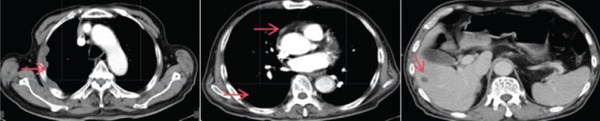
(b)

**Figure 3:**
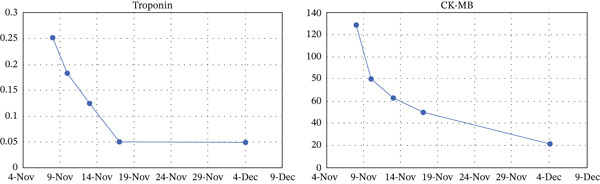
The patient′s cardiac function assessment results.

Following multidisciplinary team (MDT) evaluation, first‐line platinum‐based doublet chemotherapy combined with PD‐1 inhibition was proposed. However, given the patient′s advanced age (74 years) and family‐declined cytotoxic chemotherapy, a modified regimen was implemented: sintilimab (anti‐PD‐1 antibody, 200 mg q3w) combined with bevacizumab (anti‐VEGF antibody, 500 mg q3w). Restaging CT image after two treatment cycles demonstrated partial response (Figure 1), showing pleural lesion regression (maximal thickness: 36–16 mm) and marked reduction of metastatic lesions. At Cycle 3, Grade 2 immune‐mediated myocarditis (CTCAE v5.0) developed, characterized by elevated troponin T (0.251 ng/mL), CK‐MB (128.57 U/L), and reduced left ventricular ejection fraction (LVEF) (71%–60%). Immunotherapy was immediately discontinued, and methylprednisolone (1 mg/kg/day) was initiated. Cardiac function subsequently normalized (Figure 3), with LVEF recovering to 65%. Considering the cardio‐oncology risk/benefit balance, permanent immunotherapy withdrawal was mandated. Maintenance therapy continued with bevacizumab monotherapy (500 mg q3w). The patient ultimately passed away in February 2025, with an overall survival (OS) of 18 months from initial diagnosis.

## 3. Discussion

The SMARCA4‐UT is an exceedingly rare type of malignancy characterized by nonspecific clinical manifestations, making differentiation from other tumor types based solely on symptoms challenging. Patients typically present with a diverse array of clinical symptoms, predominantly including compressive or infiltrative manifestations. Common clinical presentations may include respiratory symptoms (e.g., shortness of breath and persistent coughing with blood‐tinged sputum), chest discomfort, and vascular complications like superior vena cava (SVC) obstruction syndrome [11]. In cases where the tumor invades the pleural tissue, patients may experience recurrent pleural effusion or pyothorax [12]. Due to the highly invasive biological nature of the SMARCA4‐UT tumor, it is not uncommon for metastases to occur early in the disease course, affecting lymph nodes, bones, the brain, or the abdominal and pelvic cavities.

Imaging studies have provided critical insights into the primary sites of SMARCA4‐UT tumors. Nambirajan et al. conducted a systematic review of CT scans from 21 patients diagnosed with SMARCA4‐UT, revealing that tumors predominantly localize in the upper and middle mediastinum [12]. These tumors frequently invade adjacent key anatomical structures, including the esophagus, bronchus, thymus, and vital vascular systems [13]. Additionally, imaging findings indicated continuous involvement of the lung parenchyma, with nearly all SMARCA4‐UT cases exhibiting aggressive and expansive growth patterns, primarily affecting the mediastinum and unilateral pleural regions, alongside extensive infiltration of mediastinal and cervical lymph nodes. Consequently, in male patients with a significant history of smoking, the presence of a large, irregularly shaped, and heterogeneously dense mass—accompanied by surrounding tissue infiltration, compressive symptoms, and signs of lymph node necrosis—should raise a high index of suspicion for SMARCA4‐UT [4, 10, 14].

The diagnosis of SMARCA4‐UTs relies on a comprehensive evaluation that includes the patient′s clinical presentation, laboratory findings, histopathological features, immunohistochemical characteristics, and genetic variant analysis. At the histomorphological level of differential diagnosis, SMARCA4‐UT may exhibit a variety of cytological patterns, such as small‐cell type, epithelioid type, or rhabdomyosarcomatous type, which can easily be confused with various neoplasms [4, 5], encompassing malignancies of diverse origins, such as hematologic neoplasms (e.g., large‐cell lymphoma), thymic and mesothelial‐derived carcinomas (thymic carcinoma and malignant mesothelioma), neuroendocrine and germ cell lineages, alongside melanocytic tumors, epithelioid sarcomas, and non–small‐cell lung carcinomas (NSCLC) (large‐cell subtype). Although immunohistochemical staining for BRG1 (SMARCA4 protein) is effective in excluding most of the aforementioned tumors, it is crucial to recognize that certain entities, such as melanoma, selected epithelioid sarcomas, large‐cell carcinomas, small‐cell lung carcinoma (SCLC), large‐cell neuroendocrine carcinomas, and epithelioid gliosarcoma‐like mesotheliomas, may demonstrate negative BRG1 expression. Melanoma, for instance, can be identified through the expression of melanocyte‐specific markers such as Melan‐A and HMB‐45. Furthermore, large‐cell carcinomas, large‐cell neuroendocrine carcinomas, and SCLC typically exhibit positive immunostaining for tight junction protein‐4 (Claudin‐4), whereas markers such as SOX2, CD34, or SALL4 are generally expressed less frequently [12–16]. In addition, SMARCA4‐deficient NSCLC—an important differential diagnosis—can often be excluded based on distinct morphological characteristics [17, 18], as its histological differentiation is usually more pronounced, frequently presenting as a well‐defined adenocarcinoma or, in rare cases, squamous cell carcinoma [19].

Pathology examination and immunohistochemistry are considered the diagnostic criteria for SMARCA4‐UTs. Based on previous studies summarizing the immunohistochemical expression profiles of this disease, Perret et al. proposed diagnostic criteria in 2019 [13], indicating that SMARCA4‐UT typically exhibits an undifferentiated morphology characterized by epithelioid, rounded, or rhabdomyoblastic tumor cells. Immunohistochemical staining typically demonstrates negativity for SMARCA2, Claudin‐4, and SMARCA4, whereas SOX2, CD34, and SALL4 are often expressed either focally or diffusely. If the pathology results do not definitively indicate SMARCA4‐UT, genetic analysis may be warranted for further confirmation of the diagnosis. In the case presented, the patient was an elderly male with a long history of smoking, and the tumor primarily involved the pleura, with secondary involvement of the mediastinum and lungs. Pathological examination revealed features consistent with poorly differentiated or undifferentiated malignancy, with a differential diagnosis that excluded squamous cell carcinoma, adenocarcinoma, large‐cell carcinoma, and other common thoracic malignancies. Immunohistochemical analysis indicated that the tumor was SMARCA4‐negative (with a positive internal control) and CD34‐positive, leading to the conclusion of SMARCA4‐UT.

As a highly malignant and recurrent tumor type, treatment options for SMARCA4‐UT have not been standardized [20]. Common treatments cover surgery, chemotherapy, radiotherapy, as well as immunotherapy and anti‐angiogenic therapy, which have received much attention in recent years [20, 21]. Targeted therapies and epigenetic treatments are also receiving considerable attention.

Due to the high malignancy and invasiveness associated with SMARCA4‐UTs, patients are frequently diagnosed at advanced stages of the disease, resulting in the loss of the opportunity for radical surgical intervention [1, 4]. Even when surgical options are available, the prognosis often falls short of expectations. Nevertheless, patients who undergo surgical resection generally exhibit a more favorable prognosis compared with those who do not; however, no statistically significant differences in OS and progression‐free survival (PFS) have been observed [21].

In terms of chemotherapy, commonly used agents include taxanes, cisplatin, etoposide, and others; their overall efficacy tends to be poor, with patients receiving chemotherapy alone typically surviving less than 1 year [1]. In contrast, immunotherapy has demonstrated more favorable efficacy in some retrospective studies and case reports, particularly in patients with high PD‐L1 expression and high tumor mutational burden (TMB) [22, 23]. Immunotherapy not only effectively prolongs patient survival but also significantly enhances their quality of life. It is important to note that immunotherapy may trigger adverse reactions in some patients; for instance, the patient in this case experienced significant immune‐mediated myocarditis. Nevertheless, the overall prognosis for patients receiving immunotherapy is more optimistic. Furthermore, studies have shown that immune agents can be effective in both high‐ and low‐immune expression patients, although the efficacy is more promising in those with high expression [1, 7, 22, 23]. Despite the encouraging results of PD‐1/PD‐L1 inhibitors in treating SMARCA4‐deficient tumors, further data are needed to substantiate these findings, particularly concerning patient selection and the optimization of combination therapy regimens.

In the realm of targeted therapies, no specific agents have been identified for the SMARCA4 gene [1]. The functional loss of the SWI/SNF complex, along with the deletion of the SMARCA4 gene [2], may result in chromatin structural anomalies that can disrupt gene expression and cellular differentiation, leading to a condition characterized by “epigenetic instability”. This mechanism is believed to play a significant role in the pathogenesis of SMARCA4‐deficient tumors (SMARCA4‐UT).

Several targeted agents, including EZH2 inhibitors, CDK4/6 inhibitors, ATR inhibitors, KRAS inhibitors, and AXL inhibitors, as well as various epigenetic modulators, have demonstrated potential therapeutic effects. These therapeutic strategies may enhance the survival and prognosis of patients with SMARCA4‐deficient undifferentiated tumors (SMARCA4‐UT) in the future [24–27]

For this patient, we implemented a treatment regimen that combined antivascular agents with immunotherapy. Although the literature suggests that the combination of antivascular agents and immunotherapeutic agents may enhance survival outcomes in patients with SMARCA4‐deficient undifferentiated tumors (SMARCA4‐UT) [17], specific efficacy data has yet to be published. In this patient, who presented with widespread metastatic disease, the follow‐up results indicated a survival duration exceeding 14 months, significantly surpassing the average OS of 4–7 months typically associated with this disease. This notable extension of survival not only provides tangible clinical benefits but also offers preliminary evidence supporting the use of antivascular agents in combination with immunotherapy for this condition. Furthermore, compared to chemotherapy combined with immunotherapy, antivascular agents exhibit a lower incidence of adverse events, thereby enhancing patient safety and tolerability. Nonetheless, further clinical data and experience are required to ascertain whether this combination therapy can be established as a standard treatment option for SMARCA4‐UT. Although emerging therapies such as immunotherapy, targeted therapies, and antivascular agents provide hope for improved patient prognosis, the management of SMARCA4‐UT continues to present numerous challenges.

## 4. Conclusion

SMARCA4‐UT represents an uncommon yet markedly aggressive malignancy characterized by poor histological differentiation, most frequently observed in mid‐adulthood populations with a documented tobacco exposure history. Its diagnosis usually relies on a comprehensive consideration of clinical manifestations and pathological features, characterized by undifferentiated tumor cells and deletion of SMARCA4. Despite the poor prognosis of the disease, immunotherapy has shown some efficacy in some patients. However, more research data are needed to verify whether the combination of immunotherapy and antivascular drugs can significantly improve the efficacy. Meanwhile, future studies should focus on exploring the molecular mechanisms of the disease and finding more effective treatment options, with the aim of improving the quality of survival and overall prognosis of patients.

## Author Contributions

Qingyang Wen and Mengle Long designed the study and supervised the overall project. Feng Li and Jiarong Yi participated in collecting data; Yaya Peng did laboratory examinations and data curation; and Di Wang and Yongjun Wu participated in data collecting and analysis. Qingyang Wen and Mengle Long contributed equally to this work.

## Funding

This work was funded by the Natural Science Fund of Science and Technology Department of Hunan Province, 2025JJ81123 and the Hunan Provincial Health Commission, D202303107662.

## Consent

Written informed consent was obtained from the patient for publication of this case report and any accompanying images. A copy of the written consent is available for review by the Editor‐in‐Chief of this journal.

## Conflicts of Interest

The authors declare no conflicts of interest.

## Data Availability

The data that support the findings of this study are available on request from the corresponding author.
